# Independent variant analysis of *TEAD1* and *OCEL1* in 38 Aicardi syndrome patients

**DOI:** 10.1002/mgg3.250

**Published:** 2017-01-25

**Authors:** Bibiana K. Y. Wong, Vernon R. Sutton, Richard A. Lewis, Ignatia B. Van den Veyver

**Affiliations:** ^1^ Department of Obstetrics and GynecologyBaylor College of MedicineHoustonTexas; ^2^ Jan and Dan Duncan Neurological Research InstituteTexas Children's HospitalHoustonTexas; ^3^ Department of Molecular and Human GeneticsBaylor College of MedicineHoustonTexas; ^4^ Department of MedicineBaylor College of MedicineHoustonTexas; ^5^ Department of PediatricsBaylor College of MedicineHoustonTexas; ^6^ Department of OphthalmologyBaylor College of MedicineHoustonTexas

**Keywords:** Aicardi syndrome, candidate genes, *OCEL1*, *TEAD1*

## Abstract

**Background:**

Aicardi syndrome is a severe neurodevelopmental disorder characterized by infantile spasms, typical chorioretinal lacunae, agenesis of the corpus callosum, and other neuronal migration defects. It has been reported recently that de novo variants in *TEAD1* and *OCEL1* each may cause Aicardi syndrome in a single individual of a small cohort of females with this clinical diagnosis. These data were interpreted to suggest that the clinical diagnosis of Aicardi syndrome may be genetically heterogeneous.

**Methods:**

To investigate this further, we sequenced *TEAD1* and *OCEL1* coding regions using DNA from 38 clinically well‐characterized girls with Aicardi syndrome.

**Results:**

We did not detect the previously reported or any other deleterious variants in any of the analyzed samples.

**Conclusions:**

This suggests that the published variants represent either an extremely rare cause of Aicardi syndrome or an incidental finding.

## Introduction

Aicardi syndrome (OMIM 304050) is a severe sporadic neurodevelopmental disorder that was characterized initially by a triad of signs: agenesis or dysgenesis of the corpus callosum, distinctive chorioretinal lacunae, and infantile spasms (Aicardi et al. 1965; Donnenfeld et al. 1989; Aicardi 1999, 2005). However, it is now recognized to be a more complex pleiotropic disorder with a spectrum of neurological and peripheral manifestations. In addition to agenesis of the corpus callosum, there are heterotopias, polymicrogyria (each in nearly 100%), intracranial cysts, cerebellar abnormalities, and severe and often intractable complex seizures (in >95%) (Taggard and Menezes 2000; Aicardi 2005; Hopkins et al. 2008). Eye abnormalities in girls with Aicardi syndrome also include optic nerve defects and acrophthalmia (Fruhman et al. 2012). Nonneuronal findings, such as costovertebral defects, predisposition to rare tumors, mild facial dysmorphism, and skin abnormalities, are also found in a smaller but substantial fraction of patients (Sutton et al. 2005; Sutton and Van den Veyver 2014).

This disease is diagnosed almost exclusively in females, with a few reported cases in 47,XXY males (Hopkins et al. 1979; Glasmacher et al. 2007; Shetty et al. 2014). Our group has demonstrated previously the excess skewing of X‐inactivation in females with Aicardi syndrome, suggesting that X‐linked gene(s) are involved in Aicardi syndrome phenotypes (Eble et al. 2009). We further assessed copy‐number variants (CNVs) in subjects with Aicardi syndrome by genome‐wide array comparative hybridization (CGH) and concluded that Aicardi syndrome is not caused by CNVs detectable with the high‐resolution array platform we used (Yilmaz et al. 2007; Wang et al. 2009).

Although Aicardi syndrome was described first in 1965 (Aicardi et al. 1965), the cause of this disorder remains uncertain. A recent publication identified de novo mutations in two affected girls: one carried a nonsense mutation in *TEAD1* (OMIM 189967) and the second harbored a missense mutation in *OCEL1* (Schrauwen et al. 2015). Therefore, this report was the first to suggest that mutations in these autosomal genes may be pathogenic and contribute to the retinal phenotypes in Aicardi syndrome. Since these variants in *TEAD1* and *OCEL1* have been reported thus far in single subjects, here, we analyzed an independent and larger cohort of 38 girls with Aicardi syndrome to confirm and assess the frequency of variants in *TEAD1* and *OCEL1* as potential causes of this disorder.

For this study, we designed primers targeting the coding exons and at least 20 nucleotides of flanking intronic sequences of both *TEAD1* and *OCEL1* and we amplified these regions by polymerase chain reactions (PCR) for Sanger sequencing and variant analyses (Appendix S1, Table S1).

Samples from 38 clinically well‐characterized girls with Aicardi syndrome were included in this study (Sutton et al. 2005; Hopkins et al. 2008; Eble et al. 2009; Fruhman et al. 2012). The relevant phenotypic features that supported the Aicardi syndrome diagnosis for these individuals are summarized in Table S2. Most subjects are of European decent (68%), and have the classic triad of phenotypes: seizures (71%), agenesis or dysgenesis of the corpus callosum (84%), and chorioretinal lacunae (74%). Twelve girls (32%) in the cohort exhibited all the common characteristics of Aicardi syndrome (chorioretinal lacunae, seizures, agenesis/dysgenesis of the corpus callosum, gray matter heterotopias, and polymicrogyria). In addition, 24 subjects (63%) exhibit additional ophthalmological phenotypes, including optic nerve abnormalities (coloboma, severe dysplasia, agenesis, atrophy, glial proliferation, pseudoadenomatous proliferation of retinal pigment epithelium, or posterior staphyloma) and microphthalmia. Also, DNA from 26 girls had been evaluated previously for X‐chromosome inactivation (XCI), and 31% of those showed skewed XCI (Table S2) (Eble et al. 2009).

We performed Sanger sequencing of the coding regions of both *TEAD1* and *OCEL1* in DNA from the included 38 subjects and were unable to identify the previously published variants (Schrauwen et al. 2015). The variants that we observed are all documented polymorphisms with high allele frequencies noted in the dbSNP database (Table 1). Nevertheless, in patients where known variants were observed, we Sanger‐sequenced the parental DNA samples where available; in all cases, the variants were inherited from one of the parents, as expected from the allelic frequencies of these single‐nucleotide polymorphisms (SNPs; Table 1). Therefore, no de novo variants were observed in either *TEAD1* or *OCEL1* in this cohort of patient samples.

**Table 1:** All variants identified in *TEAD1* and *OCEL1* are documented in dbSNP

Gene	GRCh37/hg19 coordinate	Ancestrial allele/variant allele	Documented in	dbSNP ID	Allelic frequency	Amino acid change	Patients	Mothers	Fathers
*TEAD1*	Chr11:12,923,434	T/C	dbSNP	rs2289436	T (65.2%); C (34.8%)	N/A (intron)	3/38 (7.89%)	0/11 (0%)	1/1 (100%)
*TEAD1*	Chr11:12,785,718	T/C	dbSNP	rs72858140	T (98.2%); C (1.8%)	N/A (intron)	1/38 (2.63%)	1/13 (7.69%)	0/3 (0%)
*TEAD1*	Chr11:12,903,443	C/T	dbSNP	rs2304733	C (56.6%); T (43.4%)	p.Asp171=^a^	7/38 (18.4%)	6/18 (33.3%)	3/5 (60.0%)
*OCEL1*	Chr19:17,337,025	C/T; T/T	dbSNP	rs2288544	C (77.8%); T (22.2%)	N/A (5′ UTR)	16/38 (42.1%)	5/10 (50.0%)	N/D
*OCEL1*	Chr19:17,337,223	T/C	dbSNP	rs2288542	T (70.5%); C (29.5%)	N/A (intron)	20/38 (52.6%)	6/10 (60.0%)	N/D
*OCEL1*	Chr19:17,337,280	C/T	dbSNP	rs10426390	C (92.2%); T (7.8%)	N/A (intron)	2/38 (5.26%)	1/10 (10.0%)	N/D
*OCEL1*	Chr19:17,337,281	G/A	dbSNP	rs10424828	G (94.8%); A (5.2%)	N/A (intron)	2/38 (5.26%)	1/10 (10.0%)	N/D
*OCEL1*	Chr19:17,337,447	C/T	dbSNP	rs10426800	C (76.2%); T (23.8%)	N/A (intron)	12/38 (31.6%)	4/10 (40.0%)	N/D
*OCEL1*	Chr19:17,337,555	C/A	dbSNP	rs3745163	C (77.1%); A (22.9%)	p.Thr41=^b^	13/38 (34.2%)	5/10 (50.0%)	N/D
*OCEL1*	Chr19:17,337,557	G/T	dbSNP	rs10425488	G (92.3%); T (7.7%)	p.Arg42Leu^b^	3/38 (7.89%)	1/10 (10.0%)	N/D
*OCEL1*	Chr19:17,337,871	T/C	dbSNP	rs891204	T (76.9%); C (23.1%)	p.Gly105=^b^	15/38 (39.5%)	4/10 (40.0%)	N/D
*OCEL1*	Chr19:17,337,882	C/G	dbSNP	rs891203	C (81.0%); G (19.0%)	p.Ala109Gly^b^	15/38 (39.5%)	4/10 (40.0%)	N/D
*OCEL1*	Chr19:17,337,928	C/T	dbSNP	rs1045201	C (92.1%); T (7.9%)	p.Ala124=^b^	3/38 (7.89%)	1/10 (10.0%)	N/D
*OCEL1*	Chr19:17,339,112	G/A	dbSNP	rs14129	G (77.0%); A (23.0%)	p.Lys222=^b^	15/38 (39.5%)	4/10 (40.0%)	N/D
*OCEL1*	Chr19:17,337,447	C/T	dbSNP	rs10426800	C (76.2%); T (23.8%)	N/A (intron)	13/38 (34.2%)	4/10 (40.0%)	N/D

N/A, not applicable; N/D, not done.

a NP_068780.2.

b NP_078854.1

The recent publication by Schrauwen and colleagues examined DNA from 10 girls with Aicardi syndrome and their parents. They suggested that identified de novo variants in two autosomal genes, *TEAD1* and *OCEL1*, are putatively pathogenic in Aicardi syndrome (Schrauwen et al. 2015). This was the first study to identify any potential genetic association for this disorder, but since the variants described were found only in single individuals (Schrauwen et al. 2015), we posed the question about whether either *TEAD1* or *OCEL1* variants are a more common or a rare cause of Aicardi syndrome, or whether they represent an incidental finding unrelated to the clinical diagnosis.

We attempted to verify these findings in an unrelated second cohort of diligently characterized subjects with Aicardi syndrome, who have many of the neurological and ocular characteristics associated with the condition (Table S2) (Aicardi 2005; Glasmacher et al. 2007; Hopkins et al. 2008; Fruhman et al. 2012). Our sequencing data did not detect either of the published variants in *TEAD1* or *OCEL1*. Collectively, in the samples from the 48 girls with Aicardi syndrome evaluated (10 from Schrauwen et al. and 38 from the cohort used in this study), the reported *TEAD1* Chr11:12904591G>A (NM_021961.5:c.618G>A; NP_068780.2:p.Trp206Ter) variant is found in only one subject and similarly the reported *OCEL1* Chr19:17338695G>A (NM_024578.1:c.499G>A; NP_078854.1:p.Ala167Thr) variant (Schrauwen et al. 2015) was found in only one subject. We further mined our previously published CGH data (Wang et al. 2009) and did not identify any CNVs involving either *TEAD1* or *OCEL1*. Thus, we are unable to confirm the published de novo variants in this patient cohort, and mutations in these two autosomal genes are unlikely to be a major cause of Aicardi syndrome and potentially could represent incidental findings, but clarification will await discovery of the major cause of Aicardi syndrome.

Although *TEAD1* and *OCEL1* may be rare causes of Aicardi syndrome, it is also possible that these genes may contribute to phenotypes that overlap with characteristics of Aicardi syndrome and that the phenotypic characterization of Aicardi syndrome requires refinement. A comprehensive evaluation of the described individuals with *TEAD1* and *OCEL1* variants by clinicians experienced with the diagnosis of Aicardi syndrome would also be helpful to distinguish any subtle phenotypic anomalies that might be specific to subjects harboring variants in these autosomal genes.

The *TEAD1* gene encodes for TEA domain transcription factor 1 or SV40 transcriptional enhancer factor 1 (TEF‐1), a DNA‐binding protein that acts as a ubiquitous transcriptional enhancer (Kitagawa 2007; Sawada et al. 2008; Landin Malt et al. 2013; Yuan et al. 2015). Interestingly, *TEAD1* is highly expressed in the retina and a nonsynonymous mutation in this gene has been identified previously to cause Sveinsson's chorioretinal atrophy (Fossdal et al. 2004). Sveinsson's chorioretinal atrophy, also known as helicoid peripapillary chorioretinal dystrophy, is an autosomal dominant disease characterized by symmetrical lesions radiating from the optic disk involving the retina and the choroid (Fossdal et al. 1995, 2004). Although the clinical presentation of Sveinsson's chorioretinal atrophy classically includes atrophy of the retina, retinal pigment epithelium (RPE), and choroid, the radial and serpiginous retinal and peripapillary atrophy is distinctly different from the broad, multicentric, punched out lacunae seen in Aicardi syndrome (Brazitikos and Safran 1990; Jonasson et al. 2007). The missense mutation in *TEAD1* found in patients with Sveinsson's chorioretinal atrophy is predicted to disrupt binding to YAP62, a cotranscriptional factor, and to alter the binding to downstream genes, thus affecting their transcriptional regulation (Fossdal et al. 2004; Kitagawa 2007). The nonsense mutation in *TEAD1* found in the single subject of Aicardi syndrome similarly is predicted to affect transcription of downstream targets (Schrauwen et al. 2015). A recent publication further reported that a SNP in *TEAD1* (rs926971) may influence the size of the retinal microvasculature (Jensen et al. 2016). Therefore, it is conceivable that variants in *TEAD1* may contribute to the ocular and chorioretinal anomalies observed in a rare subset of girls with Aicardi syndrome.

Unlike *TEAD1*, the function of *OCEL1* is unknown. However, *OCEL1* is highly expressed in the eye, retina, and the brain – regions that are most affected in girls with Aicardi syndrome. Interestingly, *OCEL1* is also overexpressed in mouse retina with high intraocular pressure (Panagis et al. 2011), suggesting that the regulation of this gene is responsive to ocular insults or anomalies. So far, *OCEL1* variant has only been found in one subject with Aicardi syndrome, and therefore, variant in *OCEL1* is also likely to be a rare genetic cause in Aicardi syndrome.

Since Aicardi syndrome is a disease diagnosed primarily in females and a few in 47,XXY males (Shetty et al. 2014), and excessive skewing of XCI has been shown in patients with Aicardi syndrome (Eble et al. 2009), our primary hypothesis is that the causative mutation is likely to reside on the X chromosome or that the mutation affects the X chromosome by a trans mechanism. Thus far, no studies have identified pathogenic variants on chromosome X that may be associated with Aicardi syndrome. Even though our results suggest that variants in *TEAD1* and *OCEL1* may not be major contributors to Aicardi syndrome and could each represent an incidental finding, future studies should not discount these and other autosomal genes, as they may influence the regulation of genes on the X chromosome and/or are subject to epigenetic marks in a sex‐specific manner. Additionally, based on the lack of success in identifying a single causative gene with large effect in Aicardi syndrome, this disorder may be caused by the additive effect of defects in more than one gene collectively. Therefore, for the future, it will be important to select cohorts with well‐defined phenotypes for enhanced genomic analyses, rather than comparing phenotypically heterogeneous patients, as this may augment detection of variants with potentially smaller effects. In conclusion, this thorough mutation analysis of *TEAD1* and *OCEL1* with an independent Aicardi syndrome cohort suggests that the previously published variants in these genes are either rare causes of Aicardi syndrome or an Aicardi syndrome‐like disorder or possibly incidental findings. To answer this question, large‐scale genomic efforts to search for the cause(s) of Aicardi syndrome that include larger cohorts of systematically characterized subjects must continue.

## Conflict of Interest

The authors declare no conflict of interests.

## Supporting information


**Appendix S1**. Supplemental materials and methods.

**Table S1**. Primer and PCR information.
**Table S2**. Clinical characteristics of 38 subjects with Aicardi syndrome assessed in this study.
