# Supplementary material for: Independent variant analysis of TEAD1 and OCEL1 in 38 Aicardi syndrome patients
Source: Mol Genet Genomic Med. 2017 Jan 25;5(2):117–21. doi: 10.1002/mgg3.250 (PMC5370232; doi:10.1002/mgg3.250)
Supplement: Supplementary file 1 — Appendix S1. Supplemental materials and methods. [file MGG3-5-117-s001.docx]

**Supplemental Materials and Methods**

**Ethical Compliance**

This study was conducted under a protocol approved annually by the Baylor College of Medicine Institutional Review Board for Human Subject Research.

**Subject Recruitment and DNA Preparation**

Subjects with clinically confirmed Aicardi syndrome and one or both of their parents, where available, were enrolled in this study. Clinical information and imaging results pertinent to the diagnosis of Aicardi syndrome were collected and stored in a secure database. Venous blood samples were collected for establishment of permanent lymphoblastoid cell lines from which DNA was extracted for this study. Total genomic DNA was extracted by the Puregene DNA extraction kit (Gentra Systems, Inc., Minneapolis, MN) according to the manufacturer’s protocol.

**Primer design, PCR conditions and Sanger sequencing**

*TEAD1* (NM_021961.5) and *OCEL1* (NM_024578.2) coding sequences were downloaded from NCBI for primer design. Primer sets were designed spanning each coding region including at least 20 nucleotides of intronic sequences with the Primer3 web software ([Rozen and Skaletsky, 2000](#_ENREF_1)). Fifty nanograms of genomic DNA were amplified by EmeraldAmp^®^ GT PCR Master Mix (Takara Clontech, Mountain View, CA) under standard PCR conditions. Primer sequences and annealing temperatures for each assay is detailed as Supplemental Table S1. PCR products were sent to GENEWIZ for Sanger sequencing and results were aligned to reference sequence from the GRCh37/hg19 build and analyzed with Sequencher 5.0 (GeneCodes Corporation, Ann Arbor, MI).
