# Supplementary material for: Independent variant analysis of TEAD1 and OCEL1 in 38 Aicardi syndrome patients
Source: Mol Genet Genomic Med. 2017 Jan 25;5(2):117–21. doi: 10.1002/mgg3.250 (PMC5370232; doi:10.1002/mgg3.250)
Supplement: Supplementary file 2 — Table S1. Primer and PCR information. Table S2. Clinical characteristics of 38 subjects with Aicardi syndrome assessed in this study. [file MGG3-5-117-s002.docx]

Table S1 - Primer and PCR information

Table S2 - Clinical characteristics of 38 subjects with Aicardi syndrome assessed in this study
